# Green Synthesis of Flower-Like Carrageenan-Silver Nanoparticles and Elucidation of Its Physicochemical and Antibacterial Properties

**DOI:** 10.3390/molecules28020907

**Published:** 2023-01-16

**Authors:** Syafiqah Syazwani Jaffar, Suryani Saallah, Mailin Misson, Shafiquzzaman Siddiquee, Jumardi Roslan, Wuled Lenggoro

**Affiliations:** 1 Biotechnology Research Institute, Universiti Malaysia Sabah, Jalan UMS, Kota Kinabalu 88400, Sabah, Malaysia; 2 Faculty of Food Science and Nutrition, Universiti Malaysia Sabah, Jalan UMS, Kota Kinabalu 88400, Sabah, Malaysia; 3 Institute of Engineering, Tokyo University of Agriculture and Technology, 2-24-16 Nakacho, Tokyo 184-8588, Japan

**Keywords:** silver nanoparticles, silver nanoflower, green synthesis, carrageenan, antibacterial

## Abstract

Herein, we report the green synthesis of flower-like carrageenan-silver nanoparticles (c-AgNPs) through a facile hydrothermal reaction at 90 °C for 2 h. The reduction of silver nitrate (AgNO_3_) to c-AgNPs was evident by the colour change of the solution from colourless to dark brown and further confirmed by a UV-Vis surface plasmon resonance (SPR) peak at ~420 nm. The FTIR spectra showed that the abundance of functional groups present in the carrageenan were responsible for the reduction and stabilisation of the c-AgNPs. The XRD pattern confirmed the crystalline nature and face-centred cubic structure of the c-AgNPs, while the EDX analysis showed the presence of a high composition of elemental silver (85.87 wt%). Interestingly, the morphological characterisations by SEM and FE-SEM revealed the formation of flower-like c-AgNPs composed of intercrossed and random lamellar petals of approximately 50 nm in thickness. The growth mechanism of flower-like c-AgNPs were elucidated based on the TEM and AFM analyses. The c-AgNPs displayed promising antibacterial properties against *E. coli* and *S. aureus*, with zones of inhibition ranging from 8.0 *±* 0.0 to 11.7 ± 0.6 mm and 7.3 ± 0.6 to 9.7 ± 0.6 mm, respectively, as the concentration of c-AgNPs increased from 0.1 to 4 mg/mL.

## 1. Introduction

Nanotechnology is a rapidly expanding field due to the diverse applications of nanomaterials in fields such as biotechnology, biomedicine, optoelectronics, pharmaceuticals, cosmetics, food, material science, and agriculture [1,2,3]. Silver nanoparticles (AgNPs) are one of the most fascinating metal nanomaterials that have received considerable attention in recent years because of their distinctive physicochemical properties and remarkable antibacterial activities [4,5]. The synthesis of AgNPs can be achieved via chemical and physical routes, but the hazardous effects of their by-products on the environment and the high manufacturing cost are major concerns [6,7,8]. Hence, various biomolecules, including yeast, bacteria, fungi, and algae, as well as plant extracts, have been explored for the green synthesis of AgNPs [3,9,10,11,12,13]. Plant-mediated syntheses are used more often than other biomolecules because of their easy accessibility, biocompatibility, cost effectiveness, and excellent stability under challenging experimental settings [3,4,12]. The active compounds responsible for the reduction process, on the other hand, are uncertain, and the process is relatively complicated and time consuming. Using biopolymers with a defined structure is imperative for obtaining AgNPs using the green synthesis method [14].

Polysaccharides have been identified as promising candidates for stabilising and regulating the size of AgNPs [15,16]. Thanks to the multiple binding sites along the polysaccharide chain, AgNPs can effectively be attached and trapped, thus providing significant protection against aggregation. In this case, the polysaccharide serves a dual role, as a stabiliser and a reducing agent [17]. This kind of approach enables the AgNPs to be produced using natural, cheap, and biocompatible materials. Carrageenan is a sulphated linear polysaccharide extracted from marine red algae. It is composed of D-galactose residues linked by (1→3)-linked *β*-D-galactopyranose and (1→4)-linked *α*-D-galactopyranose [14,18,19]. This polysaccharide displays a wide range of pharmacological effects, including antitumour and antiviral activity, owing to its low molecular weight, high water solubility, and a certain degree of sulphation [14]. Carrageenan also serves multiple functions in the food industry, including as gelling, emulsifying, and stabilising agents. The application of carrageenan in the synthesis of metallic nanoparticles including AgNPs is facilitated by its negatively charged surface, which contains carboxyl, hydroxyl, and ester sulphates groups, which can easily interact with positively charged metal ions via electrostatic attraction [20]. 

Several studies have shown that carrageenan can be utilised to mediate the green synthesis of AgNPs using various methods, including microwaves [21], sonochemical [22], and hydrothermal [23,24]. Most studies, however, use kappa carrageenan, which requires an additional step to isolate it from refined carrageenan using the gelling method [25]. Furthermore, the shape of the synthesised AgNPs is mostly spherical, and the application is primarily focused on dye degradation [24,26] and colorimetric sensing [27], with little information on its antibacterial effect. It has been reported that nanoparticles with novel shapes exhibit optical, electrical, and catalytic properties that are distinct from those of conventional spherical shapes, as well as wider biological and medical applications [28]. In this regard, numerous studies have focused on creating intricate Ag nanostructures [16,29,30,31,32]. Among them, flower-like AgNPs, also known as Ag nanoflowers, are particularly appealing, as their novel and collective physicochemical properties, which are not visible at the level of individual particles, can be manifested from their anisotropic structure [16]. Nevertheless, research on the synthesis of these nanostructures using biological approaches is still limited.

Herein, we report, for the first time, a facile and green method for the synthesis of flower-like AgNPs using carrageenan as a reducing and capping agent. The synthesised product, termed as carrageenan-AgNPs (c-AgNPs), was characterised using various analytical instruments including Fourier Transform Infrared (FTIR), X-Ray Diffraction (XRD), Energy-Dispersive X-Ray (EDX), and Dynamic Light Scattering (DLS) to elucidate their physicochemical properties. A Scanning Electron Microscope (SEM), Field-Emission Scanning Electron Microscope (FE-SEM), Transmission Electron Microscope (TEM), and Atomic Force Microscope (AFM) were used to observe the c-AgNPs’ morphology and to understand the growth mechanism. To investigate the antibacterial activity, disk diffusion assays were performed using *E. coli* and *S. aureus* as representatives of Gram-negative and Gram-positive bacteria, respectively.

## 2. Results and Discussion

### 2.1. Green Synthesis of c-AgNPs

**Figure 1:**
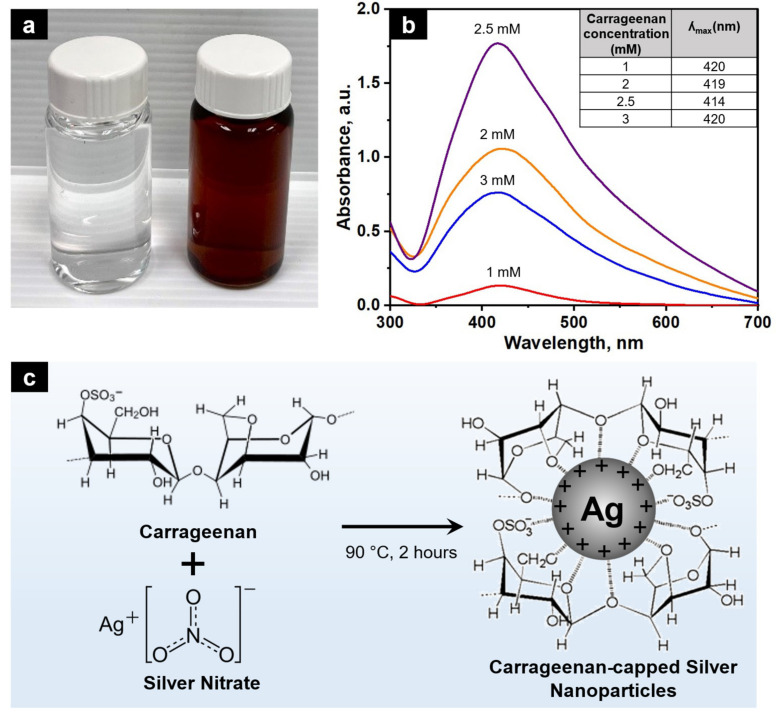
(**a**) Colour of the AgNO_3_ and carrageenan mixture before (left) and after (right) reaction at 90 °C for 2 h; (**b**) UV-Vis spectrum of AgNPs produced with different carrageenan concentrations and the corresponding ʎ_max_; (**c**) proposed mechanism of the green synthesis of AgNPs by carrageenan as a reducing and stabilising agent.

In the present study, the green synthesis of c-AgNPs was performed using a simple hydrothermal reaction with AgNO_3_ as the precursor and carrageenan solution with concentrations ranging from 1 to 3 mM as reducing and stabilising agents. The colour change of the reaction mixture was monitored, and the representative image (2.5 mM carrageenan) is shown in Figure 1a. Initially, the reaction mixture was colourless, which then turned brown after 2 h of reaction at 90 °C. This colour change was caused by the reduction of the Ag^+^ present in the precursor solution of AgNO_3_ to the atomic Ag^0^. The UV-Vis spectrum, shown in Figure 1b, confirmed the formation of AgNPs with broad bell-shaped absorption bands at 380–460 nm due to the excitation of the longitudinal surface plasmon resonance (SPR) of the AgNPs in the colloid [19]. Wan et al. [24] asserted that the water-soluble components of the carrageenan polysaccharide that are rich in hydroxyl and carboxylic groups are responsible for the effective reduction of metal cations and the stabilisation of nanoparticles (Figure 1c). Sulphate groups in carrageenan also play a vital role in the reduction process [23].

The UV-Vis spectrum also shows that the carrageenan concentration has a significant effect on the AgNPs’ formation. By increasing the carrageenan concentration from 1 to 2.5 mM, the intensity of the SPR peak also increased, which indicated an increase in the AgNP concentration, while the ʎ_max_ (wavelength at maximum absorbance) shifted to a lower wavelength (420–414 nm). However, increasing the carrageenan concentration to 3 mM reduced the SPR peak intensity and shifted the ʎ_max_ to a higher wavelength (420 nm). A similar trend was also reported previously [24]. Particle aggregation is more likely to occur at high concentrations of carrageenan, because the functional groups of the excess carrageenan interact with one another more strongly than they do with metal ions. The decrease in the absorbance and shift in the wavelength to higher values could be an indication of particle aggregation [22,23]. Therefore, from this finding, 2.5 mM carrageenan was selected for the c-AgNPs’ synthesis.

### 2.2. Characterisations of AgNPs

#### 2.2.1. Fourier-Transform Infrared (FT-IR) Analysis

**Figure 2:**
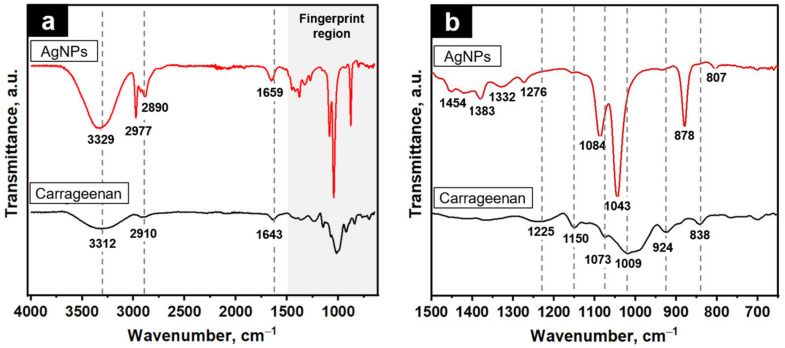
(**a**) FTIR spectra of carrageenan and c-AgNPs; (**b**) corresponding fingerprint region.

FTIR spectroscopy was used to elucidate the carrageenan and AgNPs’ chemical functionality and interaction. Figure 2a shows that the carrageenan had a broad band in the 3200–3400 cm^−1^ region due to the O-H stretching vibrations [31]. The peak appearing at ~2900 cm^−1^ is attributed to the C-H stretching vibrations of methyl groups. A typical C=O asymmetric stretching of the carbonyl groups in D-galactose was observed at 1643 cm^−1^ [27]. The fingerprint region of the carrageenan (Figure 2b) shows characteristic peaks at 1225, 1150, 1073, and 1009 cm^−1^ corresponding to the sulphate ester link vibrations. The peak observed at 924 cm^−1^ is ascribed to the 3,6-anhydro-D-galactose, while the peak at 838 cm^−1^ is attributed to galactose-4-sulfate [22]. In the case of the c-AgNPs, a narrower band was observed in the 3200–3400 cm^−1^ region, and the peak shifted to a higher wave number reveals the involvement of the O-H functional groups of the carrageenan in the c-AgNPs synthesis [24]. The shifts of the carbonyl group at 1643 to 1659 cm^−1^, the reduction in the peak intensity at 1150 cm^−1^, and the disappearance of the peak at 1225 cm^−1^, which belonged to the S=O bond of the sulphate ester groups in the c-AgNPs’ spectrum, were indicative of the participation of carbonyl and sulphate ester groups in the c-AgNPs’ formation [24]. The conjugation of the carrageenan chains onto the surface of the AgNPs was further confirmed by the presence of very strong absorption bands in the 1000–1100 cm^−1^ region, which is ascribed to the glycosidic linkage in the carrageenan [26,33]. New absorption bands in the 1250–1500 cm^−1^ region indicate an interaction between the positively charged AgNPs and the negatively charged carrageenan via Van Der Waals forces [23]. These findings suggest that the abundance of hydroxyl, carbonyl, and sulphate groups in carrageenan were responsible in the reduction and stabilisation of the c-AgNPs, in accordance with the previous reports [15,19,21,26,27].

#### 2.2.2. X-ray Diffraction (XRD) Analysis

**Figure 3:**
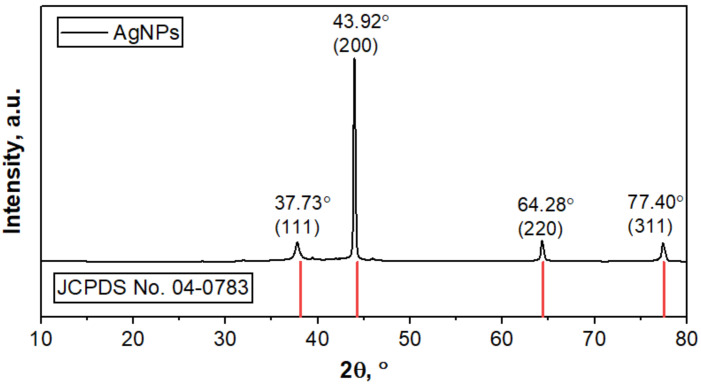
XRD spectra analysis of c-AgNPs and silver (JCPDS No. 04-0783).

The XRD pattern of the c-AgNPs in Figure 3 shows four distinct diffraction peaks at 2θ of 37.73°, 43.92°, 64.28°, and 77.40°. These peaks can be indexed to the (111), (200), (220), and (311) crystallographic planes in accordance with Bragg’s reflection of the face-centred cubic (fcc) structure of silver, as per the database of the Joint Committee on Powder Diffraction Standards (JCPDS; card number: 04-0783) [2,12]. The crystalline nature of the c-AgNPs is indicated by the intense peaks, with the peak at 2θ = 37.73° being the most intense, implying the preferred orientation along the (200) lattice plane. This finding is consistent with the one reported by Sahayaraj et al. [10], Jemal et al. [2], and Thiurunavukkarau et al. [34] for the green synthesis of AgNPs by marine algae *Padina pavonica* (Linn.), callus extracts of *Allophylus serratus*, and seaweed *Sargassum polycystum*, respectively. Based on Scherrer’s equation, the AgNPs’ crystallite size was determined to be 46.5 nm.

#### 2.2.3. Energy-Dispersive X-ray (EDX) Analysis

**Figure 4:**
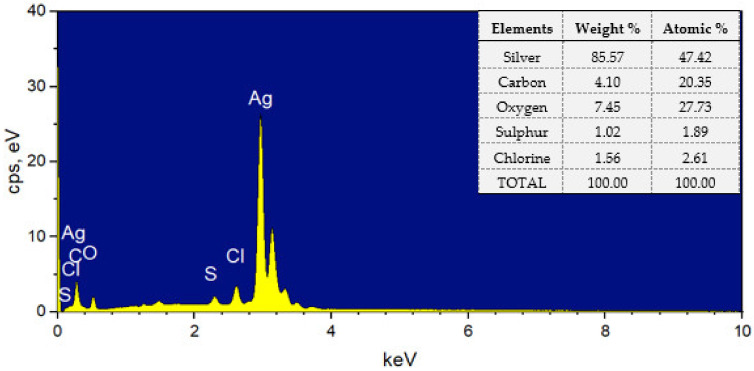
EDX spectra of c-AgNPs and the corresponding elemental composition.

The EDX pattern in Figure 4 shows the elemental composition of the sample. Generally, metallic silver has a typical optical absorption peak at approximately 3 keV due to the surface plasmon resonance. The strong Ag signal at 3 keV in the EDX spectra is indicative of AgNP formation [2,23,35]. The presence of other elements (C, O, S, and Cl) originates from organic compounds in the carrageenan that are bound on the surface of the AgNPs [23,24]. The elemental analysis revealed that the sample contained a high proportion of elemental silver (85.87 wt%). Furthermore, no signal of N from AgNO_3_ was detected, which is evidence of the successful reduction of Ag^+^ to Ag^0^ by carrageenan [13].

### 2.3. Morphology and Structural Properties of c-AgNPs

**Figure 5:**
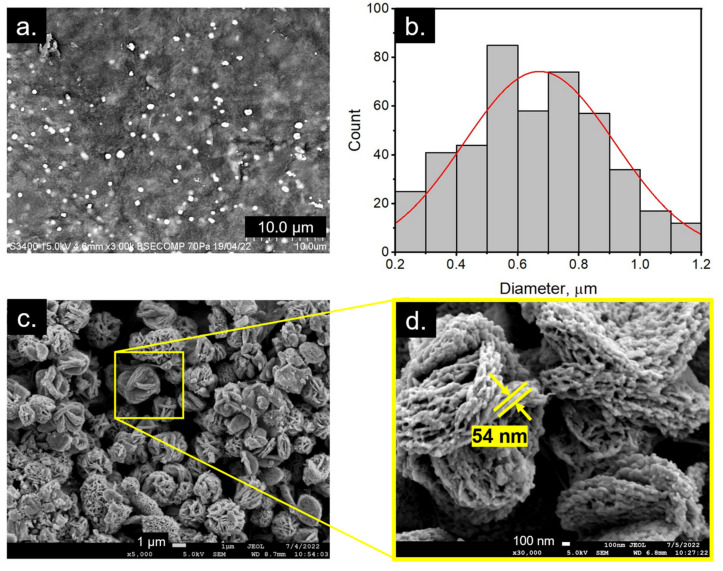
Morphological analysis of the AgNPs: (**a**,**b**) SEM image and the corresponding particle size distribution; (**c**,**d**) FE-SEM images at different magnifications.

After the synthesis, the resulting product was washed with ethanol, and its morphological structure was characterised by SEM and FE-SEM, as depicted in Figure 5. The SEM image shows that the synthesised c-AgNPs were spherical in shape, with an average size of 670 nm (Figure 5a,b). Interestingly, the high-resolution FE-SEM images revealed the flower-like details of the spherical nanoparticles, with intercrossed and random lamellar petals of approximately 50 nm in thickness (Figure 5c,d). From the UV-Vis and DLS analyses reported in the previous section, the c-AgNPs in aqueous medium were stable due to the presence of the carrageenan layer surrounding the Ag cores, which prevented them from aggregating. However, washing with ethanol and drying may cause the c-AgNPs to aggregate and form a flower-like morphology. The ethanol-induced aggregation of metallic nanoparticles has been reported in several studies [36,37]. Very recently, Nguyen et al. [30] reported the formation of wool roll-like Ag nanoflowers in an ethanol/water mixture, with sizes ranging from 280 to 700 nm. In the presence of ethanol, part of the carrageenan molecules was transferred from the aqueous phase to ethanol, resulting in the partial removal of the protective carrageenan layer on the c-AgNPs, causing aggregation [36]. Molina et al. [13] also discovered that a flower-like morphology could be obtained when AgNPs synthesised using Kalanchoe daigremontiana extract were washed with toluene and dimethyl sulfoxide (DMSO).

**Figure 6:**
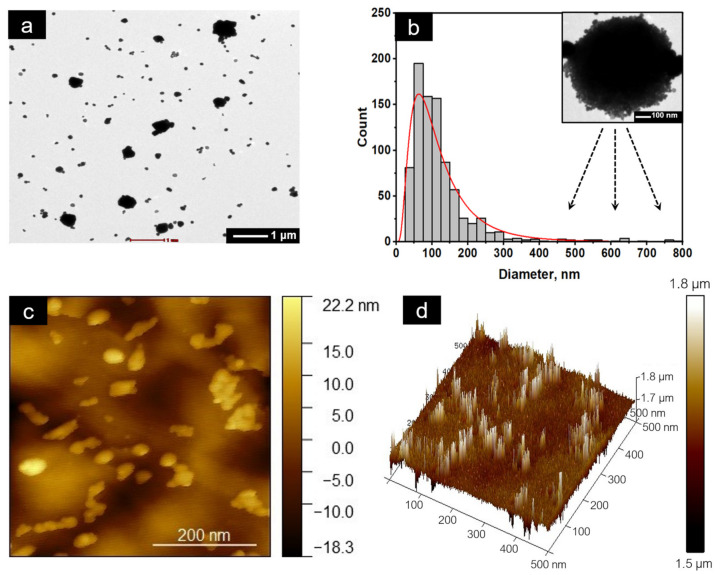
(**a**) TEM image of the AgNPs; (**b**) corresponding particle size distribution and morphology of the aggregated AgNPs; (**c**) 2D AFM image; (**d**) height analysis in 3D.

To better understand the growth mechanism, the c-AgNPs were subjected to a TEM analysis. A mixture of dispersed spherical nanoparticles and agglomerates can be observed in Figure 6a, indicating that progressive growth of the AgNPs took place, resulting in a wider particle size distribution (Figure 6b). A closer look at the agglomerated nanoparticle (Figure 6b inset) reveals the flower-like structure composed of nanoparticles with size of approximately 50 nm, consistent with the sizes obtained from the XRD analysis. This observation could be due to the formation of molecular bonds between ethanol and the c-AgNPs, which resulted in an asymmetric charge distribution on the surface of the nanoparticles. As a consequence, a dipole–dipole interaction occurred and led to the linear assembly of the nanoparticles [38], evident by the crosslinked and string-like structure observed in the FE-SEM image. Thereafter, the nanoparticles grew preferentially in specific directions, and the final product assembled into flower-like spheres composed of abundant nanopetals [16,39]. This morphology endows AgNPs with high specific activity, owing to its high anisotropy, which enhances its chemical reactivity, catalytic, biological, and optoelectronic properties [5,30].

Despite a great deal of effort to synthesise intricate Ag nanostructures including flower-like AgNPs, elucidating their growth mechanisms remains a challenge, particularly with the use of biopolymers as reducing and structure-directing agents. This is due to the presence of a large number of functional groups in the biopolymer chain compared to the common structure directing agents, such as CTAB, PVP, and citrate [16,40]. In addition to the ethanol-induced aggregation discussed above, it is possible that the carrageenan acted as a structure-directing agent that facilitated the anisotropic growth of the AgNPs into flower-like structures. The development of silver mesoflowers (AgMFs) using ascorbic acid and chitosan has been studied by Nhung et al. [16]. It was postulated that the formation of AgMFs was primarily caused by the aggregation of Ag atoms during the nucleation process, which was aided by the scrolling action of chitosan chains. The formed seeds acted as a focal point for capturing free Ag atoms from the solution, resulting in the crystallographic growth of hierarchical AgMFs. Following that, other free Ag atoms continued to diffuse toward the hierarchical structure and were deposited on the empty surfaces of the branches, eventually forming AgMFs with thicker branches and rough surfaces.

The topographical scanning of the sample using AFM, shown in Figure 6c, further confirmed the spherical nature of the polydispersed c-AgNPs, with the presence of aggregates composed of several particles forming the flower-like morphology [41,42] consistent with the FE-SEM and TEM results. The linear assembly of AgNPs was also observed. The surface roughness and height of the sample are shown in Figure 6d. Basically, the surface roughness or the texture of the surface was evaluated based on Rq and Ra [43], which denote the square root of the sum of the squares of the individual heights and depths from the mean line and the arithmetic mean of surface heights recorded across a surface, respectively [16]. With an Rq value of 70 nm and an Ra value of 55.9 nm, the c-AgNPs exhibited high surface roughness [44]. This property is especially beneficial in the development of highly sensitive Surface-Enhanced Raman Scattering (SERS) and Surface-Enhanced Fluorescence (SEF) [45]. 

### 2.4. Antibacterial Activity of AgNP-Carrageenan

**Figure 7:**
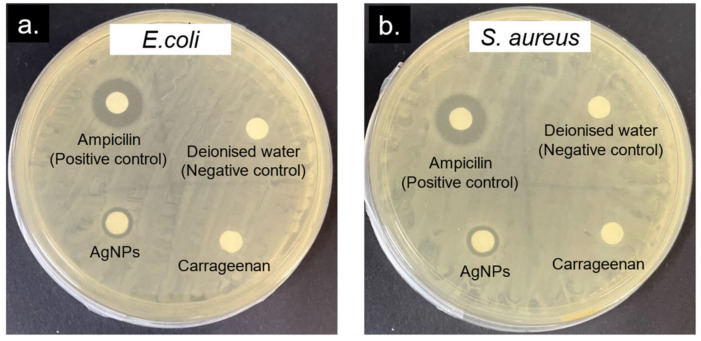
Representative agar plates of the disk diffusion assay showing the antibacterial activity of carrageenan (2.5 mM) and c-AgNPs (0.1 mg/mL) against (**a**) *E. coli* and (**b**) *S. aureus*. Ampicillin (0.1 mg/mL) and deionised water were used as the positive and negative controls, respectively.

The antibacterial activity of the c-AgNPs was tested using the disk diffusion method. *E. coli* and *S. aureus* were used as representatives of Gram-negative and Gram-positive bacteria, respectively. From the agar plates shown in Figure 7, it is observed that the c-AgNPs and standard antibiotic (Ampicilin) exhibited antibacterial activity against *E. coli* and *S. aureus*, evidenced by visible zones of inhibition. Meanwhile, no inhibition zones were observed for the deionised water (negative control) and carrageenan, implying that the samples have no antibacterial activity. 

In recent years, important advances have been achieved concerning the clarification of the antibacterial mechanism of AgNPs. However, the exact mechanism is still not completely understood [1]. In general, the antibacterial effect of AgNPs is associated to four modes of action: (1) adhesion of AgNPs onto the surface of the cell wall and membrane, (2) penetration of AgNPs inside the cell and disruption of intracellular structures (mitochondria, vacuoles, and ribosomes) and biomolecules (protein, lipids, and DNA), (3) AgNP-induced cellular toxicity and oxidative stress through the generation of reactive oxygen species (ROS) and free radicals, and (4) modulation of signal transduction pathways [46]. These actions destruct the cell morphology and result in cell death. Furthermore, the presence of Agapes and their interaction with the bacterial cell wall affects membrane permeability, thus inhibiting bacterial growth [47].

It has been elucidated that the direct contact or close proximity of the positively charged AgNPs with the negatively charged bacterial membrane significantly augmented the AgNPs’ antibacterial potential. AgNPs may cause cell wall damage, alter the transport mechanism, and impede bacterial cell colonisation. The penetration of AgNPs inside the bacterial cell promotes the formation of ROS, such as superoxide anion, hydroxyl radical, and singlet oxygen, which not only damage the cell membrane but also alter and impair the functions of biomolecules, such as proteins, DNA, and intracellular systems, thereby impeding essential metabolic processes [48,49].

**Figure 8:**
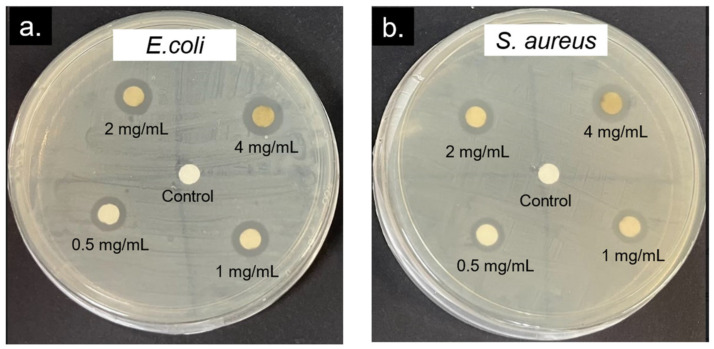
Representative agar plates of the disk diffusion assay of c-AgNPs with the concentration of 0.5–4 mg/mL against (**a**) *E. coli* and (**b**) *S. aureus*.

Figure 8 shows that the antibacterial activity of the c-AgNPs was concentration dependent, where the higher c-AgNP concentration produced a larger zone of inhibition (Table 1). This effect was more apparent for E. coli than S. aureus. The high susceptibility of Gram-negative bacteria to AgNPs compared to the Gram-positive counterpart is mainly due to the differences in the structure, thickness, and composition of the cell membrane. Several studies have shown that the presence of lipopolysaccharides (LPS) in the cell membrane causes pronounced adhesion and deposition of AgNPs onto the cell surface of Gram-negative bacteria, making them more susceptible to antibacterial therapy. Meanwhile, the negatively charged peptidoglycan that makes up the cell wall of Gram-positive bacteria is much thicker (30 nm thickness) than that of Gram-negative bacteria (3–4 nm thickness), making them more resistant to the antibacterial effects of AgNPs.

## 3. Materials and Methods

### 3.1. Materials

The carrageenan powder was purchased from EvaChem (Ampang, Selangor, Malaysia). The silver nitrate (AgNO_3_, 99.7%) was a product of Systerm (Classic Chemicals Sdn. Bhd., Shah Alam, Malaysia). The Luria Bertani (LB) agar and broth (Miller) used in the antibacterial study were supplied by Merck. The test microorganisms, *E. coli* (ATCC 25922) and *S. aureus* (ATCC 29213), were obtained from Microbiology Laboratory, Biotechnology Research Institute, Universiti Malaysia Sabah. Millipore deionised water was used throughout this work.

### 3.2. Synthesis of Carrageenan-Silver Nanoparticles (c-AgNPs)

The carrageenan solutions (1 to 3 mM) were prepared by dissolving carrageenan powder in deionised water at 90 °C under constant stirring (400 rpm) [21]. The solution was allowed to cool at room temperature before the addition of the AgNO_3_ (0.1 mM) solution at a 1:3 (v:v) ratio [22]. The mixture was heated at 90 °C for 2 h. After the reaction, the colour change of the solution was observed, confirming the c-AgNPs’ formation, which evidenced that a reduction of Ag^+^ ions to Ag^0^ took place. The solution was then kept cool at room temperature before centrifugation at 7500 rpm (25 °C) for 15 min. The suspension was separated from the solution and further washed with ethanol to dry the remaining solvent in the obtained products. The product was further dried by evaporation at room temperature to obtain solid c-AgNPs

### 3.3. Characterisations of c-AgNPs

#### 3.3.1. UV-Visible (UV-Vis) Spectroscopy

The absorbance spectrum of the AgNPs was collected using a UV-Vis spectrometer (Cary 60 UV-Vis, Agilent Technologies Inc., Georgetown, Penang, Malaysia) in a wavelength range of 300–500 nm, as characteristic peaks in this range are indicative of the formation of AgNPs. The spectrum was measured in a disposable microcuvette, with a 1 cm path length. Deionised water was used as the blank for the baseline adjustment.

#### 3.3.2. Fourier-Transform Infrared (FT-IR) Spectroscopy

Fourier-Transform Infrared (FTIR) spectroscopy analysis was used to elucidate the chemical interaction between the carrageenan and the AgNPs. The spectra were collected at room temperature using an FTIR Spectrometer (Agilent Cary 630, Agilent Technologies Inc., Danbury, CT, USA) in the infrared region between 600 and 4000 cm^−1^, with spectral resolution of 4 cm^−1^ and 32 scans.

#### 3.3.3. X-ray Diffraction (XRD) Spectroscopy

The crystallographic information of the c-AgNPs was obtained using an X-ray diffractometer (Rigaku SmartLab, Rigaku Corporation, Tokyo, Japan), operated at 40 kV and 50 mA. The c-AgNPs were scanned using a Cu-Kα radiation source with a wavelength of 1.54 Å over the diffraction angle (2θ) range of 3° to 80°. The analysis was performed in a continuous mode, with a scan speed of 4.00°/min at 25 °C. The crystallite size was calculated using the following Scherrer equation [8,15,29]:

D = kλ/βcosθ (1)

where D is the crystallite size in nm, k is a constant with a value of 0.94, λ is wavelength of the X-ray source, β is the full width at half maximum (FWHM), and θ is the Bragg angle.

#### 3.3.4. Scanning Electron Microscopy (SEM) and Field Emission Scanning Electron Microscopy (FE-SEM)

The c-AgNPs were viewed using a Scanning Electron Microscope (SEM, Hitachi S-3400N, Nara, Japan) and a Field Emission Scanning Electron Microscope (FE-SEM, JEOL JSM7900F, Tokyo, Japan) to obtain its morphological information. The sample was positioned on a stub and observed using SEM and FESEM under vacuum at an accelerating voltage of 15 and 5 kV, respectively.

#### 3.3.5. Energy-Dispersive X-ray (EDX) Spectroscopy

The elemental analysis was performed using an Energy-Dispersive X-Ray (EDX) (Bruker Nano GmbH, Berlin, Germany) connected to the SEM, with a primary energy of 15 keV. The instrument was equipped with an XFlash 5010 detector.

#### 3.3.6. Atomic Force Microscopy (AFM)

The topographical imaging was conducted using a Dimension Icon AFM instrument (Bruker, Santa Barbara, CA, USA) with a scanning speed of 0.6 line/s. Before the analysis, the diluted c-AgNP suspension was sonicated for 5 min, and a drop of the suspension was dispersed on a glass substrate and dried at room temperature.

#### 3.3.7. Transmission Electron Microscope (TEM)

The TEM imaging was conducted using a Tecnai G2 Spirit BioTWIN instrument (FEI, Hillsboro, OR, USA) with an operating voltage of 80 kV. Before the analysis, the diluted c-AgNP suspension was subjected to 5 min ultrasonication. A drop of suspension was then placed on TEDPELLA Support Films (formvar/carbon 300 mesh, copper approx. grid hole size: 63 μm) and dried at 60 °C for 20 min. The image acquired was further processed using Image J software (NIH, Bethesda, Maryland, USA) to obtain the particle size and size distribution of the AgNPs.

### 3.4. Antibacterial Activity of AgNP-Carrageenan

The antibacterial effect of the synthesised AgNPs was examined via agar the disk diffusion test method [9] using both Gram-positive and Gram-negative bacteria, *Staphylococcus aureus* (*S. aureus*) and *Escherichia coli* (*E. coli*), as the test microorganisms. Firstly, bacterial cells from glycerol stocks were streaked on agar plates and grown overnight at 37 °C to obtain single colonies. The single-colony cells were then reinoculated in Luria Bertani (LB) broth at 37 °C overnight, and the resulting bacteria cultures were used for the antibacterial study. The solution for impregnation of the disc were first prepared using ampicillin, carrageenan, and the AgNP solution. These three solutions were prepared in a sterile environment, with a concentration of 0.1 mg/mL each. The disk diffusion assay was performed on Mueller Hinton agar plates, which were first inoculated with a bacterial solution that was spread evenly on the plates using a sterile swab. Then, sterile blank discs, 6 mm in diameter, were dipped in the prepared solutions and allowed to dry before placing them on the agar plates. The antibacterial assay was conducted in triplicates, with ampicillin as the positive control and sterile water as the negative control. After all four discs were placed on the agar, the plates were incubated overnight at 37 °C. The rings formed on the agar, or the inhibition zones, were measured for each plate. The same procedures were repeated to investigate the effect of the AgNPs’ concentration on the antibacterial activity, in the range of 0.5–4 mg/mL.

## 4. Conclusions

We demonstrated a facile and green approach to the synthesis of AgNPs using carrageenan as a reducing and capping agent. The abundance of functional groups in carrageenan facilitates the formation of a protective layer on the AgNPs’ surface, resulting in the formation of c-AgNPs with a face-centred cubic structure and a high composition of silver (85.87 wt%). Upon exposure to ethanol and drying, the c-AgNPs assembled into flower-like spheres with an average size of 670 nm, composed of intercrossed and random lamellar petals of approximately 50 nm in thickness. The present work provides insight into a rapid and environmentally benign approach for the synthesis of AgNPs with a unique morphology. However, controlling the growth and size, as well as elucidating the mechanism behind the formation of flower-like AgNPs, is still a challenge and requires further investigation. In addition, the zeta potential of the AgNPs, which provides information on particle stability in aqueous suspensions, should be studied. This information is of fundamental importance in order to produce AgNPs with the defined size and structure for the intended application. The flower-like c-AgNPs with a high surface roughness may provide an opportunity for applications in printable and flexible electronics, biosensors, medical devices, and highly sensitive analytical instruments (e.g., SERS and SEF). The remarkable antibacterial properties of c-AgNPs may have prospective application as effective antibacterial agents, notably in the biomedical and food industries.

## Figures and Tables

**Table 1:** Zone of inhibition expressed in mm measured from the disk diffusion assay.

Bacteria			Zone of Inhibition (mm) *					
	Deionised Water	Carrageenan	Ampicillin (0.1 mg/mL)	c-AgNP Concentration (mg/mL)				
				0.1	0.5	1	2	4
*E. coli*	0	0	13.3 ± 0.6	8.0 ± 0.0	8.3 ± 0.6	9.3 ± 0.6	9.7 ± 0.6	11.7 ± 0.6
*S. aureus*	0	0	12.3 ± 0.6	7.3 ± 0.6	8.0 ± 0.0	9.0 ± 0.0	9.0 ± 0.0	9.7 ± 0.6

* Average value of three replicates (*n =* 3).

## Data Availability

Not applicable.

## Abbreviations

AgNPs	Silver Nanoparticles
c-AgNPs	Carrageenan-Silver Nanoparticles
AgNO_3_	Silver Nitrate
SPR	Surface Plasmon Resonance
JCPDS	Joint Committee on Powder Diffraction Standards
DMSO	Dimethyl Sulfoxide
SERS	Surface-Enhanced Raman Scattering
SEF	Surface-Enhanced Fluorescence
ROS	Reactive Oxygen Species
LPS	Lipopolysaccharides
CTAB	Cetyltrimethylammonium Bromide
PVP	Polyvinylpyrrolidone
